# Metabolic phenotyping of malnutrition during the first 1000 days of life

**DOI:** 10.1007/s00394-018-1679-0

**Published:** 2018-04-11

**Authors:** Jordi Mayneris-Perxachs, Jonathan R. Swann

**Affiliations:** 1 Unit of Nutrition and Health and Unit of Omics Sciences, Eurecat-Technology Centre of Catalonia, Avinguda Universitat 1, 43204 Reus, Spain; 2 Division of Computational and Systems Medicine, Department of Surgery and Cancer, Imperial College London, London, SW7 2AZ UK

**Keywords:** Metabonomics, Metabolomics, Metabolism, Metabolic phenotyping, Profiling, Childhood, Early-life, Undernutrition, Malnutrition, Preterm, Intrauterine growth restriction, Iron deficiency, Zinc deficiency, Environmental enteric dysfunction, NMR spectroscopy, Mass spectrometry

## Abstract

Nutritional restrictions during the first 1000 days of life can impair or delay the physical and cognitive development of the individual and have long-term consequences for their health. Metabolic phenotyping (metabolomics/metabonomics) simultaneously measures a diverse range of low molecular weight metabolites in a sample providing a comprehensive assessment of the individual’s biochemical status. There are a growing number of studies applying such approaches to characterize the metabolic derangements induced by various forms of early-life malnutrition. This includes acute and chronic undernutrition and specific micronutrient deficiencies. Collectively, these studies highlight the diverse and dynamic metabolic disruptions resulting from various forms of nutritional deficiencies. Perturbations were observed in many pathways including those involved in energy, amino acid, and bile acid metabolism, the metabolic interactions between the gut microbiota and the host, and changes in metabolites associated with gut health. The information gleaned from such studies provides novel insights into the mechanisms linking malnutrition with developmental impairments and assists in the elucidation of candidate biomarkers to identify individuals at risk of developmental shortfalls. As the metabolic profile represents a snapshot of the biochemical status of an individual at a given time, there is great potential to use this information to tailor interventional strategies specifically to the metabolic needs of the individual.

## Introduction

**Fig. 1:**
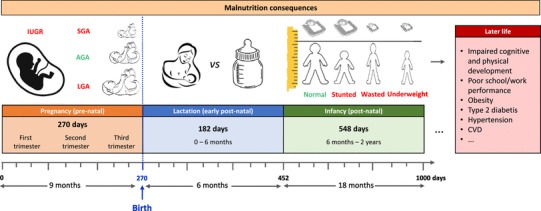
The consequences of malnutrition during the first 1000 days of life. The first 1000 days of life refers to the period from conception to a child’s second birthday. This is a critical window for rapid growth and development and nutritional abnormalities during this period can have long-term health consequences. One of the consequences of fetal malnutrition is intrauterine growth retardation (IUGR). It can also lead to infants being born small-for-gestational age (SGA), large-for-gestational age (LGA) or appropriate-for-gestational age (AGA). Other consequences of undernutrition can include children that are stunted (lower height than age-matched normal control), wasted (lower weight than age-matched normal control), or underweight (lower weight than height-matched normal control)

In the early stages of life, the so-called “first 1000 days”, nutrition has a crucial role in shaping the development and long-term health of the individual. During this period from conception until 2 years of age, nutritional deficiencies can alter the developmental trajectories of the individual leading to delayed and/or impaired immunological, cognitive and physical development. Such short-falls can be irreversible, and can lead to poor school and work achievement, and increase the risk for developing diseases later in life  (Fig. 1). In the developing world, at least 200 million children fail to achieve their developmental potential and undernutrition is a major contributing factor [1]. As such, there is a pressing need to understand the complex biomolecular perturbations induced by nutritional deficiencies during various critical windows and to identify individuals at-risk of developmental impairment. This review will discuss the potential of metabolic phenotyping for studying the biochemical consequences of undernutrition during early-life and its potential to impact on health.

Metabolic phenotyping is a systems biology approach that seeks to comprehensively assess the metabolic status of an individual. The term metabolic phenotyping encompasses both metabolomics and metabonomics. Metabolomics, refers to the measurement of a range of metabolites (the metabolome) contained within a sample, whereas metabonomics studies changes in these metabolites through time, capturing the metabolic response to perturbations or stimuli. The aim of systems biology approaches is to capture a great amount of information on a system allowing a high-resolution overview of how that system and its constitutive parts function. The term “system” can refer to a single cell, a collection of cells (tissues), a habitat, or a whole organism. The evolution of systems biology techniques has benefitted from the advancement of powerful analytical techniques capable of rapidly acquiring vast amounts of information from biological samples in a single measurement. This has coincided with increasing computing power and the evolution of statistical approaches enabling this wealth of information to be interrogated collectively rather than in isolation. This has allowed a philosophical transition away from hypothesis-led univariate comparisons towards hypothesis generating multivariate modeling. The increasing sensitivity of the analytical approaches used and the broad metabolite classes measured continually extend the resolution and minutiae at which the metabolic system can be studied. This enhanced resolution facilitates a greater understanding of the global impact of modulations to the system, regardless of their subtlety, and how this affects the overall functioning (health/disease) of the system/organism. We have previously reviewed the application of metabolic phenotyping to nutritional studies and the benefits of using this technique to model the biochemical impact of various nutritional components and the contribution of the gut microbiota [2, 3]. Here, the primary focus is on the use of this approach to characterize the temporal, chronic and systemic perturbations induced by early-life nutritional deficiencies.

## Methodological aspects of metabolic phenotyping

### Analytical methods for measuring the metabolic phenotype

Metabolic phenotyping requires analytical techniques that can simultaneously measure the abundances of many metabolites from a variety of metabolite classes (e.g., amino acids, sugars, organic acids, aromatic compounds) in a single or low number of measurements. The two main analytical platforms used to measure the metabolic phenotype are ^1^H nuclear magnetic resonance (NMR) spectroscopy and mass spectrometry (MS) (reviewed comprehensively here [4]). ^1^H NMR spectroscopy measures protons (H) on metabolites and provides semi-quantitative and structural information on a diverse range of metabolite classes (sensitivity is in the µM range). Reproducibility, robustness and simple sample preparation are major strengths of NMR spectroscopy and make this technique particularly ideal for studying large-scale sample sets (> 1000 samples). MS measures the accurate mass of molecular ions (generated from metabolites) and their relative abundances. Sensitivity is a key strength of this approach with measurements possible in the pM to µM range. As well as measuring the exact masses of the metabolite ions, MS also captures the fragmentation pattern of the ions (through MS/MS) which can be used for structural elucidation and metabolite identification. To assist with the analysis of complex mixtures, a chromatographic step usually precedes the MS analysis. This step separates metabolites based on another physico-chemical property providing additional information that is valuable for metabolite identification and improves the accuracy of quantification. The chromatographic techniques most widely used in metabolic profiling are liquid chromatography (LC; also ultra performance liquid chromatography, UPLC), gas chromatography (GC) and capillary electrophoresis (CE). The merits and pitfalls of each technique for metabolomics have been previously discussed by Lei et al. [5]. In brief, LC–MS can be used for polar or lipophilic compounds and can operate in both positive and negative ionization modes providing wide metabolome coverage. Reproducibility in peak intensity fluctuations, however, is a drawback and the lack of standardized protocols prevent consistent retention times from being obtained across laboratories obstructing the creation of standardized spectral libraries. As a result, many unknown molecular entities can be obtained from LC–MS studies. In contrast, GC–MS benefits from reproducible mass spectra that allow mass spectral library matching aiding compound identification. However, this approach requires volatile and thermally stable analytes. Many metabolites do not satisfy these criteria in their native state and so require a derivatization step prior to analysis which can introduce variability and artefacts. CE–MS is an inexpensive, fast and highly efficient separation method that is ideally suited to highly polar and ionic metabolites. However, the reproducibility, robustness and sensitivity of CE–MS is relatively poor. NMR and MS are complementary techniques that can be used independently or in parallel to provide wide metabolome coverage.

Both platforms can be used for a variety of sample types. This includes biofluids, such as urine, blood (plasma and serum), saliva, cerebrospinal fluid, milk, and fecal water as well as tissue samples. Generally, metabolites are extracted from homogenized tissue and aqueous metabolites are separated from lipophilic metabolites using liquid–liquid phase extractions prior to analysis. The biomolecular landscape can also be measured in intact tissue samples using solid-state magic-angle spinning (MAS) ^1^H NMR spectroscopy, matrix-assisted laser desorption ionization (MALDI) or desorption electrospray ionization (DESI) mass spectrometry imaging. Metabolic profiling can be performed in an untargeted manner whereby a broad range of chemical classes are measured simultaneously in an agnostic fashion. A reliance is then placed on multivariate statistical methods to extract latent information from the wealth of biochemical measures that is related to the scientific question posed. This approach is commonly referred to as hypothesis-generating as hypotheses are derived from the findings that can be subsequently tested. Targeted metabolic profiling can also be used where an analytical platform (usually MS; although NMR approaches are becoming increasingly used) is optimized to obtain highly accurate quantitative information on a single class of metabolites (e.g., bile acids [6], amino acids). Such approaches are guided by existing knowledge and can be considered both hypothesis-led and/or hypothesis-generating, due to the large number of variables measured.

### Multivariate statistical analysis of complex metabolic data

Given the wealth of data generated from these powerful analytical techniques many multivariate statistical methods have evolved to interrogate these multi- and mega-variate datasets and to integrate them with other data types (e.g., anthropometric, gut microbial, gene expression, methylation datasets) [7]. These chemometric and pattern recognition techniques can be either unsupervised or supervised in nature. The unsupervised methods include those such as principal components analysis (PCA) and hierarchical clustering analysis (HCA). These approaches work on unlabeled data, that is, they do not incorporate additional information such as sample class/groups (e.g., nourished vs undernourished) and are routinely used for an initial exploratory analysis of the data to identify trends, groupings and outliers. PCA is a projection method that seeks to summarize the multivariate data (often 10–30,000 variables) into a small number of principal components based on the largest sources of variation in the dataset. This reduction in the dimensionality of the data allows the metabolic features with the greatest variation across the dataset to be identified and the major sources of this variance to be determined. HCA clusters observations based on the similarity and differences of their metabolic profiles. Dendrograms with a distance/similarity scale are used to visualize the results generated from this method and provide an intuitive overview of metabolic similarity and distinctions. Supervised methods incorporate additional information about the samples into the models to identify metabolic variation in the data that is correlated with phenotypic response variables. Partial least squares (PLS) analysis is one of the most widely used supervised methods in metabolomics. Similar in concept to PCA, it is a projection method that captures in its components the maximum covariance between the metabolite data and the variable of interest (response/class/phenotype). When the response variables are continuous (e.g., birth weight, growth, infection burden), regression analyses (PLS) are performed and when the response variables are discrete (e.g., gender, breast-fed vs formula fed, nourished vs undernourished) discriminant analysis (PLS-DA) is used. As metabolic features uncorrelated with the variable of interest can influence the results of the PLS analysis, orthogonal projections to latent structures (OPLS) is often used. With this method, the variation in the data is separated into two blocks, one block contains the variation correlated with the variable of interest (predictive component) and the other contains the uncorrelated variation (orthogonal component). Although both PLS and OPLS have the same predictive power, the latter improves interpretation. Other approaches are now being applied to metabolic data sets, including clustering analysis methods such as K-means clustering, self-organizing maps (SOMs), and independent component analysis (ICA), for unsupervised analyses, and neural networks, support vector machines (SVM), and random forests (RF) for supervised analyses. For a comprehensive overview of statistical methods applied to these data types, readers are directed towards the following reviews [7, 8].

**Fig. 2:**
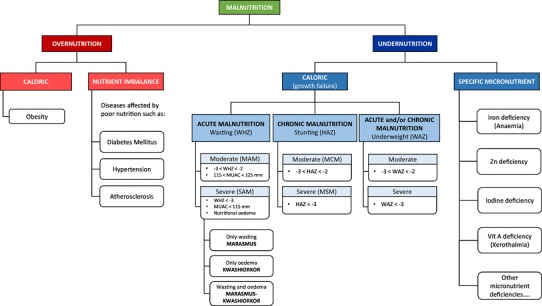
Different types of malnutrition. *HAZ* height-for-age Z-score, *WAZ* weight-for-age Z-score, *WHZ* weight-for-height Z-score, *MAM* moderate acute malnutrition, *MCM* moderate chronic malnutrition, *SAM* severe acute malnutrition, *SCM* severe chronic malnutrition

Once discriminant metabolic features have been illuminated the identification of these features is performed. This is achieved using standards, analytical software, in-house databases, and publically-available spectral libraries such as the human metabolome database (HMDB), METLIN, or Madison Metabolomics Consortium Database. Metabolites can then be grouped into pathways and functions and the biological significance of the alterations is interpreted by the investigator. Various software tools are now available to efficiently interrogate some of these databases to gain further insights into the biological pathways perturbed. The main type of analysis is enrichment analysis (also known as over-representation analysis), which identifies significantly altered metabolic pathways, and metabolite mapping, which provides a visual representation of metabolic data by showing the abundances and significances of measured metabolites in networks allowing the integration of different omics datasets [9]. A detailed overview of available tools for processing, analysis, identification, and visualization can be found in the following references [10, 11]. These approaches have been used to study the metabolic changes induced by nutritional restrictions at various phases throughout the first 1000 days of life. For this review, the prenatal and postnatal periods will be discussed separately (Fig. 2).

## Metabolic phenotyping of malnutrition during the first 1000 days of life

### Nutritional deficiencies during the prenatal period

During pregnancy, the mother is the sole source of nutrients for the developing embryo and fetus. Newborn size is the product of intrauterine growth and the supply of nutrients to the fetus is the major factor in determining this growth. Fetal nutrition is dependent upon the mother’s body size and composition, her nutrient stores and dietary intake during pregnancy and the transport of nutrients to and across the placenta. Approximately, 50% of child stunting occurs in utero and stunted mothers have a higher probability of having smaller babies than non-stunted mothers and this probability is even greater in underweight mothers [12]. Intrauterine growth retardation/restriction (IUGR) is one potential outcome of fetal undernutrition and is defined as a fetus that has failed to reach its growth potential (genetically determined potential size). It is characterized at birth by a weight and body mass lower than normal with respect to the number of gestational weeks (birth weight below the 10th percentile for gestational age and abdominal circumference under the 2.5th percentile) [13]. IUGR can result from a reduction in placental blood flow, a restriction of nutrients as well as from an excess of nutrients and hormones, or from birth defects or issues with the mother’s health. Under these conditions, the fetus may not develop appropriately and be smaller in size than normal for the gestational age [small-for-gestational age (SGA)]. In developing countries, this condition affects 6–30% of neonates while in industrialized countries the prevalence is lower (4–8%). As gestational age is not commonly known in developing countries, a low birthweight (LBW < 2500 g) is often used as an indicator for impaired fetal growth.

Numerous metabolomic studies have characterized the biochemical variation associated with IUGR in neonates (summarized in Table 1). A study by Dessi et al. used a ^1^H NMR spectroscopy-based approach to compare the urinary metabolic phenotypes of preterm neonates with IUGR with preterm neonates of suitable weight for their gestational age at birth [14]. Metabolic differences were observed between the two groups within 24 and 96 h of birth. This included greater excretion of myo-inositol, sarcosine, creatine and creatinine by the IUGR neonates compared to the controls. In response to fetal nutritional restrictions, biochemical adaptations can occur to enhance the survival chances of the fetus. This perinatal reprogramming can have adverse consequences in later life predisposing the infant to negative health outcomes. Interestingly, type II diabetes mellitus is a long-term morbidity strongly associated with IUGR and increased myo-inositol excretion has been previously associated with insulin resistance in adults. Myo-inositol is a secondary messenger in the insulin intracellular pathway and urinary myo-inositol has been proposed as a marker of glucose intolerance [15]. Such changes in IUGR infants are thought to occur due to persisting adaptations to intrauterine hypoglycemia following maternal malnutrition. Similar results were observed in a study comparing the urinary metabolic profiles of IUGR, appropriate-for-gestational age (AGA) and large-for-gestational age (LGA) newborns over the first week of life [16]. Low carbohydrate tolerance due to hypoglycemia can be seen with both fetal hyponutrition (IUGR) and hypernutrition (LGA) and in this study both the IUGR and LGA infants had similar urinary metabolic phenotypes at birth that were distinct from the AGA infants. Again, this was characterized by greater excretion of myo-inositol, as well as threonine, in IUGR and LGA compared to AGA neonates. Two additional studies reported consistent findings. Barberini et al. observed that urinary myo-inositol was the most important discriminatory metabolite between LGA/IUGR infants and AGA infants and Dessi et al. found that urinary myo-inositol, creatinine, creatine, citrate, urea, and glycine differed between groups [17, 18]. A study comparing the biochemical signatures of cord blood from pigs born either with IUGR or a normal body weight found glucose to be less abundant in the umbilical vein plasma of IUGR neonates consistent with intrauterine hypoglycemia [19]. Such changes may drive an adaptive response reflected in changes to myo-inositol metabolism, although, in these pigs no differences were found in insulin across the groups. Interestingly, the IUGR pigs had greater circulating concentrations of HDL-C, LDL-C and total cholesterol compared to those of normal birth weight indicative of modified lipid and cholesterol metabolism, which may have later-life ramifications for obesity and cardiovascular diseases.

**Table 1:** Metabolomic human studies on nutritional deficiencies during the prenatal period

Study	Population	Characteristics	Methods	Samples	Main findings
Dessi et al. (2011) [14]	Preterm newborns	IUGR (*n* = 26) AGA (*n* = 30)	^1^H NMR	Urine (24 and 96 h of age)	IUGR vs AGA ↑ myo-inositol, sarcosine, creatine, creatinine
Horgan et al. (2010) [23]	Term infants	SGA (*n* = 8) AGA (*n* = 9)	Targeted LC–MS	Placental villous explants	Significant difference between SGA and AGA when both were cultured at 6 or 20% O_2_ tensions No difference between SGA and AGA when both cultured at 1% O_2_
Favretto et al. (2012) [22]	Term infants	IUGR (*n* = 22) AGA (*n* = 21)	LC–MS	Cord blood plasma	IUGR vs AGA ↑ Amino acids (phenylalanine, tryptophan, glutamate, methionine) ↓ Kynurenine
Barberini et al. (2014) [17]	Term infants	IUGR (*n* = 11) LGA (*n* = 12) AGA (*n* = 10)	GC–MS	Urine (12 h of age)	IUGR + LGA vs AGA ↑ myo-inositol Urea, glycerol, glucose, citric acid, and uric acid were also important for clinical class discrimination
Dessi et al. (2014) [18]	Term infants	IUGR (*n* = 12) LGA (*n* = 9) AGA (*n* = 17)	^1^H-NMR	Urine (8 h, 4 and 7 days from birth)	IUGR vs AGA ↑ myo-inositol, citrate, creatinine, creatine, betaine/TMAO, and glycine ↓ Urea, aromatic compounds, and branched chain amino acids
Marincola et al. (2015) [16]	Term infants	IUGR (*n* = 8) LGA (*n* = 8) AGA (*n* = 8)	^1^H-NMR	Urine (first week of age)	IUGR vs AGA infants ↑ *myo*-inositol, threonine, glycine, creatine, creatinine, citrate ↓ succinate, betaine
Liu et al. (2016) [25]	Preterm and term infants	IUGR Preterm (*n* = 35) Term (*n* = 25) AGA Preterm (*n* = 35) Term (*n* = 25)	Targeted LC–MS (amino acids and acyl-carnitines)	Blood (3–7 days after birth)	Preterm AGA vs term AGA infants ↑ Alanine, glutamine, pipecolic acid, proline ↓ Homocysteine, heptanoyl carnitine, sebacoyl carnitine Preterm IUGR vs term IUGR infants ↑ Homocysteine, heptanoyl carnitine, decanoyl carnitine, methylmalonyl carnitine, glutaryl carnitine, sebacoyl carnitine, hydroxyacetyl carnitine, hydroxyhexadecenyl carnitine ↓ Arginine, glutamine, histidine, leucine, isoleucine, ornithine, serine, threonine, tryptophan, valine, linoleyl carnitine
Abd El-Wahed et al. (2017) [20]	Term infants	SGA (*n* = 40) AGA (*n* = 20)	Targeted LC–MS (amino acids and acyl-carnitines)	Cord blood	SGA vs AGA ↑ Several acylcarnitines including C18–OH, C16–OH ↑ Amino acids (alanine, arginine, aspartate, citrulline, glutamine, isoleucine, leucine, ornithine, phenylalanine, tyrosine, valine) ↓ Histidine, methionine

*AGA* appropriate-for-gestational age, *IUGR* intrauterine growth retardation, *LGA* large-for-gestational age, *SGA* small-for-gestational age, *TMAO* trimethylamine-*N-*oxide

A study using a UPLC–MS-based approach found clear metabolic differences in the cord blood collected shortly after birth from SGA and gestational age-matched healthy, AGA neonates [20]. SGA blood contained greater amounts of glutamine and acylcarnitines, particularly C18–OH and C16–OH acylcarnitines, and lower amounts of histidine and methionine. These changes are likely to reflect alterations in the availability of energy substrates. Alongside glucose, glutamine is a major source of cellular energy during fetal life and acylcarnitines are important for energy metabolism, being responsible for shuttling fatty acids into the mitochondria for fatty acid oxidation. While the ratio of free carnitine to total carnitine can be indicative of the nutritional status of the individual, the ratios between short-, medium- and long-chain acylcarnitines can reflect fatty acid metabolism status. Consistent with the observed differences in myo*-*inositol, modulations in plasma acylcarnitines have been previously reported with type II diabetes. For example, long-chain acylcarnitines directly interfere with insulin signaling within the cell membrane [21]. Furthermore, the pathogenesis of IUGR has been associated with the placental transport of acylcarnitines and amino acids. Another study found 22 metabolites to differ in cord blood from IUGR newborns and AGA fetuses [22]. This comprised seven amino acids including tryptophan, phenylalanine and glutamate, which were increased in IUGR samples compared to AGA. The hyper-catabolic state of IUGR neonates could underlie these observations or they may arise from alterations in placental metabolism. An in vitro study investigating placenta villous explants from neonates noted tryptophan and phenylalanine to be increased under SGA compared to AGA conditions [23]. Elevations in plasma tryptophan, a precursor for serotonin, has also been reported previously in IUGR infants [24].

Amino acids, carnitine and acylcarnitines are important for the developing fetus and are transported through the placenta from the mother to the fetus. Using a targeted LC–MS-based approach these were measured in blood collected from pre-term and term newborns either SGA or AGA 3–7 days after birth [25]. Several metabolites changed according to birth weight, including alanine, leucine, tyrosine, serine, ornithine, methionine, homocysteine, isovaleryl-carnitine and eicosanoyl-carnitine. While gender-dependent variation was not seen in AGA newborns it was present in the IUGR newborns with females having greater amounts of circulating aspartate and glutamate but lower amounts of crotyl-carnitine compared to males. Gestational age-dependent metabolic variation was also seen, with a greater number of changes noted in the IUGR infants compared to those AGA. Preterm newborns with IUGR had lower amounts of circulating amino acids (glutamate, threonine, histidine, arginine, leucine, isoleucine, valine, ornithine, serine, tryptophan) and higher amounts of different carnitines compared to their full-term equivalents. These authors speculate that lower amino acids in the preterm infants relate to impaired transport of amino acids by the placenta and that certain amino acids, including arginine and leucine, only increase late in pregnancy. Leucine and isoleucine can activate the mTOR pathway stimulating protein synthesis in skeletal muscle and promote embryonic development [19]. Given these findings, there is potential for amino acid and carnitine supplementation to attenuate the metabolic derangements of IUGR to achieve the growth potential of the fetus. These studies collectively catalogue the broad metabolic changes induced by fetal malnutrition in the infant and their potential to predispose the individual to later-life metabolic disorders.

### Early postnatal nutrition (0–6 months)

Aside from intra-uterine quality of nutrition, diet in the first weeks to months of life can determine the future health of newborns. Human milk is commonly acknowledged as the most complete form of nutrition for infants. Exclusive breast-feeding is recommended for the first 6 months of life to achieve optimal health, development and growth of infants. The composition of human milk is dynamic and is influenced by multiple factors including maternal diet, duration of pregnancy and stage of lactation [26]. The metabolic composition of breast milk from mothers of preterm and term infants has been determined over the first month of lactation using ^1^H NMR spectroscopy [27]. In total, 69 metabolites were observed to change dynamically in human milk over this period. This included 15 sugars, 11 energy-related metabolites, 23 amino acids and derivatives, 10 fatty acid-associated metabolites, 2 vitamins, 3 nucleotides and 5 bacterially-derived metabolites. Human milk oligosaccharides (HMOs) are a major component of human milk that act as prebiotics for commensal bacteria. Many of these HMOs were seen to decrease in breast milk over the first month reflecting their importance in shaping the early bacterial colonization of the neonatal gut. HMOs are complex sugars containing a lactose core bound to one or more glucose, galactose, fucose, sialic acid or *N*-acetylglucosamine residues. A group of HMOs contain α-1,2 fucosyl linkages, which include 2′-fucosyllactose (2′-FL), lactodifucotetraose (LDFT), lacto-*N*-fucopentaose (LNFP) I, or lacto-*N*-difucohexose I. These are produced through the action of fucosyltransferase 2, encoded by the secretor gene, *FUT2*. Some mothers have a mutation in the *FUT2* gene preventing them from making these linkages. These individuals are termed ‘non-secretors’ while those that are heterozygous or contain no mutations are termed ‘secretors’. As the infant gut cannot produce glycosidases necessary to catabolize these HMOs, they reach the colon where they act as decoy receptors for enteric pathogens. These HMOs in milk from secretor mothers prevent pathogens from adhering to the intestinal epithelium protecting the infant against infection and pathogen-associated diarrhea [28, 29]. A study using LC–MS, GC–MS, CE–MS and NMR spectroscopy, identified 710 metabolites in breast milk some of which changed dynamically over the first 3 months of lactation [30]. This included short-chain fatty acids (SCFA), which serve as an important energy source for the infant and help to drive the development of the gastrointestinal tract [31]. Di- and triacylglycerols increased over time in breast milk while several HMOs and phosphocholines decreased. The same authors explored the role of HMOs in Group B *Streptococcus* infections in infants from Gambia [32]. Maternal genes, including those that define the Lewis blood group antigen, regulate the expression and activity of several glycosyltransferases that synthesize HMOs in the mammary tissue. Using ^1^H NMR spectroscopy the Lewis status of the mother could be identified from the HMO signature of their breast milk. Infants of Lewis-positive mothers were less likely to be colonized by Group B *Streptococcus* at birth than those born to Lewis-negative mothers and were also more likely to clear the infection subsequently.

**Table 2:** Metabolomic human studies on malnutrition during the post-natal developmental phase

Study	Population	Characteristics	Methods	Samples	Main findings
Acute undernutrition					
Bartz el al. (2014) [46]	Ugandan children (6–59 months)	SAM (*n* = 62) WHZ < − 3 or MUAC < 110 mm or Concurrence of edema and malnutrition	Targeted LC–MS (aminoacids, acetylcarnitines) Untargeted GC–MS	Plasma	Malnourished children ↑ NEFAs, ketones, even-chain acylcarnitines ↓ Albumin and amino acids After nutritional rehabilitation ↓ NEFAs, ketones, even-chain acylcarnitines, myo*-*inositol, ethanolamine ↑ Amino acids (BCAA, alanine, ornithine, threonine, cysteine, tryptophan)
Giovanni et al. (2016) [48]	Malawian children (9–59 months)	SAM (*n* = 40) 21 kwashiorkor moderate/severe (nutritional oedema) 19 marasmus WHZ > 3 and/or MUAC < 115 mm Community controls (*n* = 157) 78 stunted (HAZ < − 2) 79 non-stunted	Targeted LC–MS/MS	Serum	Kwashiorkor vs marasmus at baseline ↓ Amino acids including aspartate, tryptophan and kynurenine ↓ Acylcarnitines, acylcarnitine / free carnitine SAM at admission vs SAM after nutrition intervention ↑ Amino acids ↑ Kynurenine, α-aminoadipic acid, asymmetric dimethylarginine SAM after nutrition intervention vs controls ↓ Amino acids ↓ Kynurenine, α-aminoadipic acid, asymmetric dimethylarginine
McMillan et al. (2017) [51]	Nigerian children (6–48 months)	SAM (*n* = 47) WHZ < − 3 and/or MUAC < 115 mm and/or Nutritional oedema Control (*n* = 11) MUAC > 12.5 mcs or WHZ ≥ − 1	Untargeted GC–MS LC–MS	Plasma Stool	SAM vs control children (only differences in plasma) ↑ Even-chain acylcarnitines, dihexoses, lactate, decanoylcarnitine, angiotensin I, a sphingoid base ↓ Amino acids (glutamine, arginine, tyrosine, BCAA, and tryptophan catabolite kynurenine) ↓ Phosphatidylcholines, phosphatidylethanolamines, oxylipins
Chronic undernutrition					
Mayneris-Perxachs et al. (2016) [59]	Brazilian children (6–24 months)	Cases (*n* = 158) WAZ < − 2 Controls (*n* = 168) WAZ > − 1	Untargeted ^1^H-NMR	Urine	Cases vs controls ↓ Betaine, DMG ↑ Gut microbial–host cometabolites (PAGn, 4-CS, 3-IS) ↑ 2-PY, NMNA NMND and BAIBA were predictors of short-term catch-up growth
Semba et al. (2016) [68]	Malawian children (12–59 months)	Stunted (*n* = 194) HAZ < − 2 Non-stunted (*n* = 119)	Targeted LC–MS/MS	Serum	Stunted vs non-stunted ↓ Essential amino acids (BCAA, methionine, threonine, histidine, phenylalanine, lysine, tryptophan) ↓ Conditionally essential amino acids (arginine, glycine, glutamine) ↓ Non-essential amino acids (asparagine, glutamate, serine) ↓ Citrulline, ornithine, taurine, dimethylarginine, carnitine and sphingomyelins
Environmental enteric dysfunction					
Farràs et al. (2018) [71]	Zambian children (6–23 months)	Hospitalised children with SAM (*n* = 20)	^1^H-NMR	Urine	Villus blunting associated with ↓ Gut-microbial metabolites (4-HPA, PAGn, 3-IS, acetamide, 4-hydroxyhippurate, DMA, TMA), energy and muscle metabolites (succinate, creatine, HMB) ↑ Sucrose
Vitamin D deficiency					
Wang et al. (2014) [92]	Chinese children (6–35 months)	Children with nutritional rickets (*n* = 112) Healthy controls (*n* = 88)	UPLC-Q-TOF-MS/MS	Urine	31 biomarkers of nutritional rickets were identified. The main pathways affected were: ascorbate and aldarate metabolism, pentose and glucuronate interconversions, taurine metabolism, calcium metabolism, and fatty acid oxidation A combination of two metabolites provided a good diagnostic for rickets ↓ Sebacic acid and ↑ phosphate
Finkelstein et al. (2015) [93]	Pregnant African American adolescents (13–18 years)	adequate vitamin D (*n* = 15) low vitamin D (*n* = 15)	GC–MS HPLC–MS/MS	Serum	Low vitamin D vs adequate vitamin D ↓ Pyridoxate, bilirubin, xylose, cholate ↑ Leukotrienes, 1,2-propanediol, azelate, undecanedioate, sebacate, complement 3 peptide, piperine

*2-PY N*-methyl-2-pyridone-5-carboxamide, *3-IS* 3-indoxyl sulfate, *4-HPA* 4-hydroxyphenylacetate, *BAIBA* beta-aminoisobutyric acid, *DMA* dimethylamine, *DMG* dimethylglycine, *HMB* β-hydroxy-β-methylbutyrate, *m-HPPS m*-hydroxyphenylpropionyl sulfate, *NMNA N-*methyl nicotinic acid, *NMND N*-methylnicotinamide, *PAG* phenylacetylglycine, *PAGn* phenylacetylglutamine, *TMA* trimethylamine, *TMAO* trimethylamine-*N-*oxide

Variability across individuals in molecules contained in milk collected 90 days postpartum was studied. In total, 65 metabolites were measured, including fatty acids, amino acids, energy metabolites, mono-, di-, and oligosaccharides, vitamins and nucleotides [33]. While some metabolites were found to vary widely across individuals, lactose, myo-inositol, glutamate, citrate, valine, creatinine, and urea were highly conserved across individuals. This indicates that these metabolites have important functional roles for the developing neonate. For example, lactose is the most abundant metabolite in breast milk. It provides ~ 40% of the total energy intake of the infant and is a major osmotic component. Although the total concentration of HMOs was tightly regulated across the individuals a large amount of variation was seen in the specific oligosaccharides present. Consistent with differences in secretor status, 2′-FL, LDFT, LNFP I and LNFP II were highly variable across mothers. Fucose was positively correlated with α1,2-linked and negatively correlated with the α1,3/4-linked fucosyloligosaccharides. Such associations imply that free fucose could reflect metabolism of α1,2-linked fucosyloligosaccharides either by human enzymes or those derived from bacteria present in the milk.

Agnoux et al. studied the impact of feeding an isocaloric low-protein (8%) diet to rats throughout gestation and lactation on milk composition using an untargeted MS approach [34]. Although perinatal protein restriction did not modify the protein content of the milk it did significantly reduce the amount of free amino acids at the end of lactation. This included several amino acids involved in gluconeogenesis and insulin secretion such as tyrosine. This could have downstream consequences for the insulinotrophic effect of milk and the growth of the offspring. Indeed, pups nursed by protein-restricted mothers had lower plasma insulin and glucose during the lactation period compared to pups nursed by well-nourished dams. In addition, an increase in milk fat concentration was observed from the first day of lactation in protein-restricted rats. The preservation of the overall protein content of the milk as well as an increase in fat content may represent an adaptive strategy by the mother to maintain the energy intake of the pup despite a reduction in milk production.

Where breast-feeding is not possible, formula milk has been developed to provide nutrients for infants. Several metabolic profiling studies have been performed to understand how breast-feeding and formula-feeding differentially impact on the metabolic state of the infant. A study by Marincola et al. compared the metabolic phenotypes of term infants fed either exclusively breast milk or formula milk during the first 4 months of life [35]. Using NMR spectroscopy, the urinary metabolic profiles showed formula-fed infants excreted greater amounts of choline, threonic acid, and pantothenate at 60 and 130 days of life than breast-fed infants. Choline is important for cell membrane integrity, lipid metabolism, cholinergic nerve function and in neonatal brain development. Pantothenate is key for the biosynthesis of fatty acids, cholesterol, acetylcholine and threonic acid is a product of vitamin C oxidation. Increased excretion of these metabolites by formula-fed infants likely reflects the higher amount of these compounds or their precursors in formula milk compared to breast milk. The excretion of energy-related metabolites (citrate, formate, *cis-*aconitate, lactate) was also found to vary with feeding practices. The long-term consequences of these differences have yet to be defined but these observations highlight how compositional variation in milk can imprint on the biochemical state of the developing infant. Dessi et al. found that the metabolic variation induced by diet (formula- versus breast-milk) was greater than the variation associated with anthropometric measures (IUGR, LGA, AGA) [36]. GC–MS was used to compare the urinary metabolic profiles at 3 and 7 days post-partum from IUGR, AGA, and LGA neonates fed either breast milk or formula milk. At day 3, breast-fed infants excreted higher amounts of aconitic acid, aminomalonic acid and adipic acid while the formula-fed infants excreted greater glycine, pseudouridine, citrate, myo-inositol and homoserine. Formula-fed infants also excreted greater amounts of glucose and galactose than those receiving breast milk, which is likely due to the greater amounts of these sugars in the formula milk.

The biochemical implications of formula- and breast-feeding practices have also been studied in an infant rhesus monkey model [37]. Both the urine and serum metabolomes of these monkeys were characterized using ^1^H NMR spectroscopy. Overall, the formula-fed infants were observed to have larger body sizes, higher blood insulin and differences in their inflammatory and metabolic profiles. Specifically, metabolic changes consistent with altered amino acid, protein and sugar metabolism were observed in the urine and differences in amino acids and ketones were noted in the serum. This included higher branched chain amino acids (BCAA) in the sera of formula-fed infants compared to breast-fed infants. This may be associated with the greater circulating insulin in these animals given that BCAA have a role in insulin secretion and have been associated with an insulin-resistant phenotype [38]. This could also contribute to the enhanced growth of these monkeys as insulin has been shown to act as a growth promoter. The cytokine profiles showed a greater amount of inflammation in the formula-fed infants compared to the breast-fed infants which was thought to be related to the immunological properties of breast milk. This inflammation could increase intestinal permeability and may explain the greater excretion of lactose in the formula-fed monkeys due to its higher absorption from the gut. Collectively, these studies highlight how nutrition during early development can modulate the biochemical status of the developing individual. The long-term effects of these feeding practices on the metabolic phenotype have yet to be defined but warrant further investigation.

### Malnutrition during the post-natal developmental phase (6 months–2 years)

According to the World Health Organization (WHO), malnutrition is defined as “the cellular imbalance between the supply of nutrients and energy and the body’s demand for them to ensure growth, maintenance, and specific functions”. As such, malnutrition encompasses both under- and over-nutrition. Undernutrition is the result of food intake that is continuously insufficient to meet dietary energy requirements while overnutrition refers to chronic intake of food that exceeds the dietary energy requirements resulting in overweight and/or obesity. In this review, we will focus solely on undernutrition. Several review articles have summarized the application of metabolomics to study obesity [39–42].

Undernutrition is a major public health problem in developing countries and is recognized as an underlying factor in ~ 45% of all child deaths [43]. Undernutrition during critical windows of early development bestows upon individuals’ lifelong consequences including stunting, impaired vaccine efficacy, impaired cognitive function, and a predisposition to obesity, diabetes and cardiovascular diseases [44]. Acute malnutrition is caused by a significant decrease in food consumption in a short period of time resulting in rapid weight loss or wasting. The weight-for-height Z-score (WHZ) and the mid-upper arm circumference (MUAC) are used to classify children as wasted. Moderately acutely malnourished (MAM) children can be defined as having a MUAC between 115 and 125 mm or a WHZ 2 standard deviations below the populations average (WHZ < − 2). Severe acute malnutrition (SAM), previously referred as protein–energy malnutrition (PEM), is associated with high mortality and is estimated to cause 1–6 million child deaths every year. Severely acutely undernourished children are defined by a MUAC lower than 115 mm and WHZ < − 3. In contrast, chronic undernutrition is a slow, cumulative process caused by a sustained inadequate intake of nutrients typically manifesting as stunted growth. The length-for-age Z-score (LAZ) or height-for-age Z-score (HAZ) is used to classify a child as stunted. Children with moderate chronic undernutrition (MCM) have an LAZ or HAZ < -2 while children with an LAZ or HAZ < -3 are classed as severely chronically undernourished (SCM). Finally, the weight-for-age Z-score (WAZ) is used to classify children as underweight. As wasting is an indicator of acute undernutrition and stunting is an indicator of chronic undernutrition, underweight reflects both acute and chronic undernutrition although it cannot distinguish between them.

UNICEF estimates that 41 million children globally are acutely undernourished and that stunting affects 800 million people worldwide with 195 million children under 5 years classified as stunted. While nutritional interventions have reduced mortality, the long-term effects of undernutrition for those that survive remains a major challenge. Understanding the biomolecular mechanisms through which undernutrition can contribute to its major clinical phenotypes and its long-term outcomes will help guide novel interventions to prevent such developmental shortfalls and reduce this societal burden. Hence, metabolic phenotyping offers promise to resolve the multitude of biochemical events that occur with nutritional deficiencies and as the metabolic system attempts to restore homeostasis.

#### Caloric undernutrition

##### Acute undernutrition

Most metabolic phenotyping studies investigating early-life undernutrition have focused on acute undernutrition in humans and various animal models (Table 2). During acute malnutrition, there is insufficient food intake to meet the energy and nutrient demands of the body. As a result, autophagy can occur where body fat and muscle is broken down resulting in wasting, the metabolic rate is reduced, kidney function is disrupted and the immune system is impaired. In acutely malnourished children the rates of death from diarrhea, malaria, pneumonia and measles are greatly increased [45]. Bartz et al. conducted one of the first comprehensive metabolomic studies to investigate the metabolic derangements associated with SAM in children from Uganda aged 6 months to 5 years [46]. Plasma metabolomes were characterized by a combination of LC–MS, targeting amino acids and acetylcarnitines, and an untargeted GC–MS-based approach. At baseline, acutely malnourished children were reported to have high circulating amounts of non-esterified fatty acids (NEFAs or free fatty acids), ketones, and even-chain acylcarnitines and low amounts of albumin and amino acids. However, a matched nourished control group was not included in this study and so it is assumed that these observations are derived from comparisons to age-matched reference values. High circulating amounts of free fatty acids, ketones and even-numbered acylcarnitines indicate that the hydrolysis of lipid stores and the oxidation of fatty acids form part of a key adaptive response to acute childhood malnutrition. In-hospital mortality remains high (10–30% of all treated cases) in children with SAM after nutritional rehabilitation following WHO-established protocols and following discharge (8.7% within 3 months of discharge [47]). These circulating metabolites were sharply reduced after the nutritional rehabilitation of these infants, as were myo-inositol and ethanolamine, consistent with a lower turnover of complex lipids. Several plasma amino acids (alanine, isoleucine, leucine, proline, valine, ornithine, threonine, cysteine, tryptophan) were also found to increase after treatment. The authors hypothesize that nutritional therapy increased the availability of dietary protein resulting in a greater oxidation of amino acids and a reduction in fatty acid oxidation. As a result, fat deposition is increased, and weight gain is promoted. Leptin, a hormonal marker of adipose tissue reserve and a modulator of immune function, was found to be a strong predictor of mortality prior to and during nutritional rehabilitation.

Two clinical phenotypes of SAM exist, marasmus and kwashiorkor with an intermediate state of marasmic-kwashiorkor. Marasmus is characterized by severe wasting (WAZ < − 3), whereas edema (swelling caused by excess fluid trapped in tissues) is characteristic of kwashiorkor. Hepatic steatosis, hypoalbuminemia and loss of hair pigmentation is also seen with kwashiorkor. As such, marasmus is considered an adaptive response to starvation while kwashiorkor is considered a maladaptive response to this stress. Typically, marasmus results from an insufficient intake of energy required to meet the body’s demands, whereas kwashiorkor can result from an insufficient intake of protein despite adequate intake of carbohydrates. To understand the pathophysiologic mechanisms underlying the clinical phenotypes of severe acute undernutrition, Giovanni et al. investigated the serum metabolomes of Malawian children aged 9–29 months [48]. These investigators used a targeted LC–MS/MS approach to measure 141 metabolites including sugars, amino acids, biogenic amines, sphingomyelins, acylcarnitines, and phosphatidylcholines. Hospitalized children with kwashiorkor and marasmus were compared to non-stunted and stunted community children at baseline and 3 days after nutritional stabilization. Metabolic distinctions were observed between the two SAM phenotypes at baseline with most metabolites (128 out of 141) being lower in sera from kwashiorkor children. This included lower circulating amounts of amino acids in children with kwashiorkor compared to those with marasmus, including the essential amino acids aspartate, tryptophan and its derivative kynurenine. These findings are consistent with inadequate protein intake with kwashiorkor and previous observations that protein breakdown in response to food deprivation is different between these phenotypes [49]. Manary et al. previously noted an accelerated rate of protein turnover in children with marasmus and acute infections compared to those with kwashiorkor and acute infections [50]. Other biochemical differences included lower acylcarnitines with kwashiorkor and a lower acylcarnitine to free carnitine ratio compared to marasmus indicative of reduced fatty acid transport into the mitochondria for β-oxidation in the kwashiorkor state. Carnitine synthesis requires lysine and methionine and both amino acids were lower in the sera of kwashiorkor children vs those with marasmus. Differences in the bioavailability of these precursors could limit the flexibility in energy strategies. Nutritional therapy was observed to improve the abundance of several metabolites in these children including circulating amino acids, biogenic amines, and lysophosphatidylcholines. Both SAM phenotypes were found to be metabolically distinct from the non-stunted and stunted community controls, even after nutritional stabilization. This included variation in the circulating abundance of amino acids, biogenic amines, sphingomyelins, acylcarnitines, and phosphatidylcholines. The persistence of these metabolic disruptions indicates the long-lasting effects of these metabolic adaptations and may contribute to the poor long-term outcomes of these individuals.

Another study applied an untargeted multi-platform (GC– and LC–MS) approach to measure the stool and plasma metabolomes of Nigerian children aged 6–48 months with SAM (21 with marasmus and 26 with kwashiorkor) and compared them to 11 hospital control children [51]. While no SAM-associated metabolic variation was identified in the fecal profiles the SAM and control children could be discriminated based on their plasma metabolomes. Consistent with the findings of Bartz et al., children with SAM exhibited a marked reduction in amino acids (glutamine, arginine, tyrosine, BCAA, and the tryptophan metabolite kynurenine) and an increase in even-chain acylcarnitines [46]. Several phospholipids belonging to the phosphatidylcholine and phosphatidylethanolamine families were significantly decreased with SAM as were several oxylipins. Oxylipins are bioactive lipids derived from the oxidation of long-chain polyunsaturated fatty acids (LCPUFA), including arachidonic acid-derived eicosanoids. These lipids perform important functions such as immune regulation, tissue repair and blood clotting. LCPUFA deficiency has been previously observed in children with SAM and is hypothesized to be a product of both dietary deficiencies and increased β-oxidation of fatty acids during starvation (indicated in this study by increased even-chain acylcarnitines) [52]. In addition, dihexoses, lactate, decanoylcarnitine, angiotensin I, free iron (III) heme, and a sphingoid base were found to be higher in the plasma of SAM children compared to nourished children. Disaccharides are not readily absorbed and increased plasma dihexoses suggest increased movement of these sugars through the intercellular space of the intestinal mucosa indicating impaired gut function with reduced intestinal barrier function. Interestingly, no differences were observed in the plasma or fecal metabolomes between edematous and non-edematous malnutrition. However, the relatively low sample numbers and wide age range may have been a factor in obscuring such differences.

The temporal biochemical impact of SAM has been studied on the plasma metabolome of juvenile pigs [53]. In this model, symptoms consistent with marasmus were induced by feeding 4-week-old weaned pigs a nutrient-deficient maize diet for 7 weeks. This diet had a comparable energy content to the reference diet but contained 61% less digestible protein. Maize-based diets are common in low-income countries and are known to be deficient in lysine, tryptophan, and vitamins A and B. Weekly profiling of the plasma metabolome using an untargeted LC–MS strategy revealed the evolution of a variety of biochemical disruptions. Circulating essential (valine, leucine, lysine, threonine, methionine, phenylalanine, tryptophan) and non-essential (glutamine, glutamate, proline, tyrosine) amino acids decreased in the undernourished pigs compared to the reference pigs. This was in-line with previous findings in marasmic children [54]. Many of these changes occurred rapidly after the first and second weeks of undernutrition and became more pronounced as the study progressed. Histidine was the only plasma amino acid observed to increase with undernutrition. Carnosine is a dipeptide of histidine and β-alanine and is present in high concentrations in skeletal muscle. Increased histidine may reflect an adaptive response to maintain or recover homeostasis through muscle degradation. This was supported by increases in pseudouridine from the first week of acute malnutrition, which is released from the catabolism of tRNA and rRNA, indicating greater turnover of muscle cells. Pseudouridine, uric acid and allantoin are metabolites involved in nucleoside metabolism and all were increased following SAM. Interestingly, uric acid, a product of purine metabolism, changes in this marasmus model but is not observed to change in kwashiorkor children [55]. This raises the potential for plasma uric acid to be a biomarker for distinguishing edematous and non-edematous SAM. Other circulating metabolites altered by SAM were those derived from the gut microbiota (hippurate, phenylacetylglycine, and *p*-cresol glucuronide) suggesting that gut microbial–host metabolic exchange is disrupted with undernutrition.

To understand the wide-reaching systemic effects of early-life protein–energy undernutrition, Preidis et al., investigated the metabolic derangements occurring in the neonatal mouse model of undernutrition [56]. In this model, protein–energy undernutrition was induced by timed separation of neonates from lactating dams. Here, 5-day-old CD1 mice were separated from dams for 4, 5, and 12 h on days 5, 6, and 7–15 days of life, respectively. Control mice had unrestricted access to nursing. Following 11 days of caloric restriction, urine, stool, plasma, ileal fluid, cecal fluid and liver tissue were collected. Untargeted profiling using a combination of GC–MS and LC–MS approaches identified 695 metabolite pairs significantly different between the experimental groups. Consistent with the initiation of autophagy, amino acid and lipid catabolites represented 22, 21, and 16 of the 30 most important urine, plasma, and stool metabolites, respectively. Similarly, undernourished mice also had greater amounts of monoacylglycerols and free fatty acids in the liver and greater amounts of carnitine in the urine than their nourished equivalents. Carnitine conjugates (isobutyrylcarnitine, 2-methylbutyrylcarnitine, isovalerylcarnitine) derived from the catabolism of BCAA were also present in higher amounts in the urine, plasma and liver of the undernourished animals. All bile acids were found in lower concentrations in the undernourished mice except for tauromuricholate (plasma) and taurolithocholate 3-sulfate (ileum, cecum, and colon). The metabolite with the greatest fold-change in stool was the secondary bile acid, deoxycholic acid, which was present in lower amounts in the undernourished feces. Bile acids can inhibit the growth of intestinal bacteria, including pathogenic species, and may influence the susceptibility of the host to enteric infections [57]. Metabolites indicative of inflammation and oxidative stress, features commonly observed in undernourished infants, were altered across different sample types with undernutrition. Collectively, this study highlights the profound impact of early-life undernutrition on the biomolecular landscape of the individual and the global attempts of the metabolic system to adapt and compensate for nutrition insufficiencies. This same model was also used to investigate whether the metabolic derangements were resolved after refeeding and catch-up growth [58]. In total, 287 metabolites (98 in urine, 73 in plasma and 116 in stools) differed in relative concentrations between undernourished and control mice immediately after nutrient restriction. The most significant group of metabolites were microbial-derived products of aromatic amino acid metabolism. Plasma concentrations of the tryptophan derivative, serotonin were lower in undernourished mice, whereas several tryptophan catabolites (5-hydroxyindoleacetate, indolelactate, and *C*-glycosyltryptophan) were increased in the urine compared to control animals. Following 3 weeks of refeeding the deficits in body weight and length of the undernourished mice had recovered. Similarly, the urinary and plasma metabolomes also became more comparable to the nourished profiles with only 20 urinary and 15 plasma metabolites differing between the two groups at this sampling point. Despite this systemic improvement and catch-up growth, the fecal metabolome and the intestinal microbiota of the undernourished mice remained markedly distinct from the control mice. After refeeding, the metabolites that remained most disrupted were the aromatic amino acid phenylalanine and the derivatives of aromatic amino acids, phenyllactate, and *p*-cresol sulfate.

The detailed metabolic effects of early-life protein deficiency on the urinary metabolome of mice has been resolved by ^1^H NMR spectroscopy [59]. In this model, C57Bl6 mice were weaned onto a protein deficient (2% protein, 84.2% carbohydrates) diet and compared to mice weaned onto a standard diet (20% protein, 64% carbohydrates). Following 13 days of protein deficiency, an up-regulation of the TCA cycle was observed, indicated by the higher excretion of TCA cycle intermediates, *N*-methylnicotinamide, and nicotinamide-*N*-oxide. The higher carbohydrate content of the protein-deficient diet (84.2%) compared with the standard chow diet (64.1%) may be a factor driving these alterations. Intriguingly, and consistent with observations in children and animals, undernutrition was also found to modify the functional capacity of the gut microbiome. Several amino acid catabolites derived from the gut microbiota were excreted in lower amounts compared to the nourished mice. These catabolites included isobutyrate, isovalerate, polyamines (cadaverine and putrescine), and BCAA catabolites (2-oxoisocaproate, 2-methyl-2-oxovalerate, 2-oxoisovalerate). The gut microbial metabolites of choline (dimethylamine, trimethylamine, and trimethylamine-*N*-oxide) were observed to increase in the urine of protein malnourished mice, with choline itself reduced compared to the nourished mice, indicative of enhanced microbial choline utilization following protein deprivation. Other microbial–mammalian co-metabolites that were disrupted by the protein deficient diet included 2-hydroxyisobutyrate, hippurate, phenylacetylglycine (PAG), 4-hydroxyphenylacetate, *m*-hydroxyphenylpropionylsulfate, and cinnamate derivatives. To further explore the impact of protein deficiency on the community structure of the gut microbiota, microbial profiling using 16S rRNA gene sequencing was performed on stool samples collected at various times of protein deficiency. The microbial and metabolic profiles were then integrated to identify alterations in the cross-talk between the intestinal microbes and the host following protein malnutrition. From these analyses protein deficiency was observed to restrict the maturation of the gut microbiome as well as its functionality and biochemical exchange with the host. Given the known importance of the gut microbiota on host health, including indigestion and the gut–brain axis, these alterations could have profound effects on host development.

As a significant component of mammalian biocomplexity and an influential factor on host metabolism, digestion and immunological maturation, microbial–host metabolic cross-talk has been investigated further. Changes in the gut microbiota were explored in the CD1 neonatal mouse model of undernutrition. In these animals, protein–energy undernutrition reduced the diversity of the intestinal microbiota and depleted the microbiome of genes that extract energy from non-digestible components [60]. However, no differences were observed in the fecal microbiota of Nigerian children with SAM compared to nourished hospital controls and no fecal metabolites derived from the gut microbiota were observed to change [51]. The limited sample size in this study may have prevented such differences from being detected. To further investigate the role of the gut microbiota in the pathogenesis of acute undernutrition Smith et al. transplanted kwashiorkor-associated fecal microbiota from children into C57BL/6J germ-free mice [61]. In this model, stool samples were collected from twin pairs discordant for kwashiorkor (healthy well-nourished twin vs kwashiorkor twin). Both groups were freely fed a regional Malawian diet (low caloric density, nutrient-deficient diet) for 21 days and compared to their respective controls fed a standard mouse chow diet. Targeted GC–MS of SCFA and untargeted GC–MS metabolomic analyses were performed on fecal and cecal samples, while untargeted ^1^H NMR spectroscopic analyses were used to measure the urinary and plasma metabolic profiles of the mice. The combination of the Malawian diet and the kwashiorkor microbiome resulted in marked weight loss in the recipient mice compared to the mice receiving the healthy co-twin microbiota on the same diet. These mice excreted higher amounts of allantoin (an end-product of purine metabolism in non-primates, equivalent to uric acid in humans), creatine and creatinine (markers of muscle turnover) and gut microbial-derived metabolites of choline (methylamine, dimethylamine, and trimethylamine-*N*-oxide) and amino acid metabolism (PAG, 3-indoxyl sulfate, and hippurate). In addition, fecal methionine and cysteine (involved in sulfur metabolism) concentrations were significantly lower in mice harbouring the kwashiorkor microbiota compared with those carrying healthy co-twin microbiota when consuming a Malawian diet. Selective inhibition of the TCA cycle by the kwashiorkor associated gut microbiota was also observed. These findings support the notion that the gut microbiota participate in the pathogenesis of SAM.

##### Chronic undernutrition

The urinary metabolic phenotypes of Brazilian children aged 6 months–2 years enrolled in a case–control study were characterized using ^1^H NMR spectroscopy [62]. In this study, similar metabolic associations were found with all measures of stunting (HAZ), underweight (WAZ) and wasting (WHZ). Stunted children excreted lower amounts of betaine and dimethylglycine, endogenous metabolites of choline. These changes indicate that choline and betaine bioavailability is lower in chronically undernourished children. As these are important methyl donors such differences may have downstream implications for various functions reliant on methylation including epigenetic processes such as DNA methylation. In addition, the gut bacterial-host co-metabolites of the amino acids phenylalanine (PAG), tyrosine (4-cresyl sulfate), and tryptophan (3-indoxyl sulfate) were excreted in greater amounts by the stunted children suggesting a functional modulation of the gut microbiome towards increased proteolytic activity. In addition to 3-indoxyl sulfate, other metabolic perturbations reflected disruptions to tryptophan metabolism. The majority of tryptophan is metabolized to kynurenine via the hepatic enzyme tryptophan 2,3-dioxygenase (TDO) or the enzyme indoleamine 2,3-dioxygenase (IDO), which is expressed in several extra-hepatic tissues. While TDO protects against toxic levels of tryptophan accumulating in plasma, IDO is increased in response to infection and inflammation [63]. *N*-methyl-2-pyridone-5-carboxamide (2-PY) and *N*-methylnicotinic acid are downstream metabolites of kynurenine and were excreted in higher amounts by stunted children. This indicates greater oxidation of tryptophan to kynurenine in these individuals. The downstream metabolites of kynurenine suppress the proliferation of activated T cells, effectively dampening the immune response to prevent the pathology associated with chronic inflammation [64]. These changes are most likely in response to systemic inflammation as a result of increased intestinal permeability and persistent infections. This is supported by observations in these same children where the plasma kynurenine: tryptophan ratio was positively correlated with plasma levels of lipopolysaccharide-binding protein [65]. Research in Peruvian and Tanzanian children also found plasma tryptophan and kynurenine to be associated with systemic markers of inflammation, growth and oral vaccine response [66]. This metabolic adaptation to inflammation and enhanced microbial-utilization, coupled to reduced dietary intake of tryptophan and malabsorption, depletes the availability of tryptophan for the host. Tryptophan is an essential amino acid and is necessary for protein synthesis and growth, immunomodulation and the biosynthesis of the neurotransmitter, serotonin [67]. In this study, *N-*methyl-nicotinamide (NMND) and β-aminoisobutyric acid (BAIBA) were also identified as metabolic predictors of catch-up growth 6 months later in stunted children. NMND was positively associated with future growth while BAIBA was inversely associated with growth. Through different mechanisms, both metabolites reflect energy sparing strategies by the individual allowing greater energy availability for growth. Identification of such biomarkers may provide useful tools for detecting children at risk of further growth shortfalls allowing interventional strategies to be targeted towards those most in need.

Another study used a targeted approach to measure serum amino acids in children from rural Malawi [68]. Here, stunted children aged 1–5 years had lower serum concentrations of a variety of amino acids including essential (isoleucine, leucine, valine, methionine, threonine, histidine, phenylalanine, lysine), conditionally essential (arginine, glycine, glutamine), and non-essential amino acids (asparagine, glutamate, serine), as well as citrulline. As with the Brazilian, Peruvian and Tanzanian children, tryptophan was also found to be lower in the stunted children. Additional changes included lower serum ornithine, taurine, dimethylarginine, carnitine and six sphingomyelins in stunted compared to non-stunted children.

The metabolic effects of long-term protein–energy malnutrition have been studied in Sprague–Dawley rats through 5 weeks of strict dietary restriction (deprived to 60% of the amount consumed by control rats) [69]. Long-term starvation resulted in a range of biochemical derangements including alterations to the TCA cycle (*cis*-aconitate, citrate, isocitrate, succinate), choline metabolism (betaine), and purine metabolism (uric acid). It also caused an increased excretion of metabolites involved in carnitine (3-hydroxyisovalerylcarnitine), muscle (creatine, creatinine) and tryptophan/nicotinamide metabolism (kynurenic acid, 2PY/4PY, dihydroxyquinoline). These findings were consistent with many of those seen in children and highlight the wide range of metabolic processes modulated by chronic nutritional restrictions.

##### Environmental enteric dysfunction

Environmental enteric dysfunction (EED), previously termed environmental enteropathy or tropical sprue, is a condition characterized by villous atrophy, chronic inflammation of the small intestinal mucosa, malabsorption and increased intestinal permeability. It is highly prevalent in South America, South Asia and sub-Saharan Africa and arises from the additive effects of a malnourishing diet and chronic exposure to enteric pathogens. This condition is considered a significant driver of childhood stunting, cognitive impairment and metabolic derangements that predispose individuals to later-life disorders such as obesity, cardiovascular diseases and diabetes. These have recently defined as “*HAZdrop*”, *“COG-hit”* and “*Met-syn”*, respectively [70]. As a major hallmark of EED, metabolites associated with villus damage were investigated in hospitalized children with SAM from Zambia [71]. In this study, measures of villus health obtained from small intestinal biopsies were correlated with matched urinary metabolic phenotypes. Villus blunting was associated with a reduced excretion of many gut microbial-derived metabolites (4-hydroxyphenylacetate, phenylacetylglutamine, 3-indoxyl sulfate, acetamide, 4-hydroxyhippurate, dimethylamine, trimethylamine), as well as metabolites related to energy and muscle metabolism (succinate, creatine, β-hydroxy-β-methylbutyrate). The lower excretion of these metabolites is likely explained by malabsorption from the gut but decreases in the gut bacterially-derived metabolites could also stem from the reduction in epithelial surface area in the gut, and therefore, inhabitable niches for the microbes. In these children, sucrose was found to be excreted in significantly higher amounts by the two children with the shortest villi. In a healthy gut, sucrose is hydrolyzed by sucrases in the brush border to glucose and fructose. Increased excretion of sucrose with EED suggests that this enzyme activity is reduced and that paracellular absorption occurs due to a loss in barrier function. In agreement with this, sucrose excretion was positively correlated with the lactulose: rhamnose excretion ratio in these children.

Using a combination of malnourishing diets and enteric infections common to developing countries various rodent models of EED have been developed. The double hit of protein malnutrition and *Cryptosporidium* infection in the mouse was found to amplify the biomolecular disruptions induced by protein deficiency alone and impact on a variety of biochemical processes [72]. This included fluctuations in the energy-related pathways, microbial choline degradation, tryptophan metabolism, and biomarkers of inflammation and oxidative stress. The parasite also modified the community structure of the fecal microbiota. Interestingly, many of the infection-associated metabolic changes were not correlated with the microbiome suggesting that these may be products of parasite metabolism. Another study by Brown et al. found several metabolic changes in the small intestinal contents of mice fed a malnourishing diet (7% protein and 5% fat) compared to those on a nourished diet (20% protein and 15% fat) [73]. In total, 420 metabolites differed between the two groups including dramatic shifts in the small intestinal bile acid pool (reduced tauro-conjugated bile acids in the malnourished mice). Pathways involved in linoleic acid metabolism, and the biosynthesis of phenylalanine, tyrosine, tryptophan, BCAA, and bile acids were all significantly under-represented in the malnourished animals while steroid and sphingolipid biosynthesis pathways were over-represented compared to their nourished counterparts. Furthermore, differences were noted in many vitamins in the small intestine. Consistent with other studies, malnutrition modulated the bacterial community structure of the small intestine, which assisted the acquisition of environmentally acquired microbes. The authors demonstrated that coupling this malnourishing diet with a set of commensal Bacteroidales and *E. coli* strains synergistically triggered physiological changes consistent with EED. This included villus blunting and exacerbated the impact of the malnourishing diet on inflammation, intestinal permeability, and growth stunting.

#### Micronutrient deficiencies (hidden hunger)

Micronutrient deficiencies occur when the intake and absorption of vitamins and minerals are insufficient to sustain health and development. Such deficiencies are also known as ‘hidden hunger’ because their effects are not typically obvious until the deficiency becomes severe. Micronutrient deficiencies are mostly described in terms of iron, zinc, iodine, and to a lesser extent vitamin A, as these deficiencies are the most common and lead to visible clinical symptoms. Their effects are particularly devastating in the first 1000 days of life and each year these are estimated to cause 1.1 million of the 3.1 million child deaths that occur due to undernutrition [43, 74]. Even modest deficiencies in these vitamins and minerals can have notable effects on development and health, including physical and cognitive consequences. Poor diet, infections, and disease are all factors that can contribute to micronutrient deficiencies. Several metabolic phenotyping studies have been performed to detail the biomolecular disruptions arising from such nutritional limitations (Table 2).

##### Iron deficiency

Iron deficiency (ID) is the most common micronutrient deficiency in the world, affecting 3.5 billion people worldwide. Children under 3 years are one of the most affected groups and preterm infants and infants born to mothers with certain gestational complications (IUGR, malnutrition) are particularly vulnerable to becoming iron deficient early in infancy [75]. Iron is an essential nutrient because iron cofactors activate enzymes involved in most of the major metabolic processes in the cell. This includes biological processes such as respiration, energy production, DNA synthesis, and cell proliferation. Iron is also important for neurotransmission, synaptogenesis and myelination during brain development. ID can result in iron deficiency anemia (IDA), where there are insufficient healthy red blood cells to carry adequate oxygen to the body’s tissues, or it can persist without progression to anemia (non-anemic ID). IDA has been shown to cause long-term behavioral and cognitive impairments despite starting iron treatment soon after the diagnosis of anemia [76]. Non-anemic ID is threefold more common than IDA and can also result in long-term neurodevelopmental effects [77, 78]. Currently, most infants are screened for ID at 1 year of age by measuring hemoglobin. However, the risks of ID to the developing brain are greatest in the period prior to testing, particularly during the fetal and early neonatal phase. As such, there is a need to identify ID and children at risk of ID earlier in life.

Some studies have used in vivo ^1^H NMR spectroscopy [magnetic resonance spectroscopy (MRS)] to track the metabolic consequences of ID on the developing brain in early life. For example, Carlson et al. determined whether hippocampus specific ID impaired memory systems in mice by genetically inducing 40% iron reduction in late fetal life [79]. Although the ID mice had comparable striatal iron content compared to their iron sufficient controls, striatal glucose and lactate were reduced while phosphocreatine and the phosphocreatine/creatine ratio was increased. These changes are indicative of altered energy metabolism, consistent with the significance of iron in these processes. Rao et al. used a diet-controlled model of ID to evaluate the effect of fetal and neonatal ID on metabolite concentrations in the developing hippocampus of rat pups [80]. Perinatal ID was induced by feeding the pregnant dam an ID diet from gestational day (GD) 3 to post-natal day (PD) 7, followed by an iron-supplemented diet. These pups were compared to those born to iron sufficient dams that were fed an iron-supplemented diet throughout the experiment. Hippocampal metabolites were assessed from PD 7 to PD 28. As with the genetic model, ID resulted in changes to brain energy metabolism (elevated phosphocreatine, glutamate, and phosphocreatine/creatine ratio with ID), as well as neurotransmission (elevated aspartate, glutamate, taurine, GABA with ID), and myelination (elevated *N-*acetylaspartate with ID). To gain further insight into the long-term effects of perinatal ID, the same authors conducted a study using this model of perinatal ID, but rat pups were weaned at PD 21 onto an iron-supplemented diet [81]. At PD 56, the hippocampal metabolic profile was quantified. Despite the recovery of iron concentrations in the brain at 8 weeks, the neurobiochemical profiles of the rats remained distinct from their iron sufficient counterparts. Glutamine was higher in the ID brains and creatine, lactate, *N*-acetylaspartate-glutamate and taurine were lower compared to the iron sufficient profiles. This demonstrates that ID-induced alterations in energy metabolism, neurotransmission and myelination remain despite the resolution of iron deficiency. Similarly, Ward et al. studied the long-term effects of early-life ID on the metabolome of the developing striatum and striatum-dependent behaviors of rats, after ID resolution [82]. ID was achieved through the administration of a low-iron diet from GD 3 to PD 7, a minimally iron-supplemented diet from PD 7 until weaning on PD 22, and an iron-supplemented diet until PD37. Consistently, the biochemical changes reflected disruptions to energy metabolism (phosphocreatine, creatine, glucose, lactate), amino acids, neurotransmitters (glutamate, GABA, and taurine), and markers of neuronal and glial integrity (glycerophosphocholine, phosphocholine) and myelination (glutamine, myo-inositol, and *N*-acetylaspartate) at PD 22. However, in contrast to the findings of Rao et al., despite the persistence of behavioral abnormalities, most of the metabolome changes were resolved by PD 37 when the concentration of iron in the brain approached control values.

The impact of early-life IDA on the metabolic composition of cerebrospinal fluid (CSF) from rhesus monkeys was assessed using a ^1^H NMR spectroscopy-based metabolomic approach [83]. Suppression of TCA cycle activity in the intrathecal compartment was indicated during the anemia period by lower citrate: pyruvate and citrate: lactate ratios and a higher pyruvate: glutamine ratio in the IDA infants compared to the iron sufficient animals. However, these metabolic changes were lost following the resolution of IDA suggesting that these CSF alterations could be normalized following the recovery of iron status. Using a similar approach, the long-term effects of non-anemic ID were studied in the CSF of monkeys over the first year of life [84]. As with the IDA monkeys, the CSF from the non-anemic ID monkeys contained higher pyruvate: glutamine ratios and lower phosphocreatine: creatine at 4 months of age, indicative of impaired TCA cycle activity prior to the presentation of anemia. The lower phosphocreatine: creatine ratio was also in agreement with the perturbations observed in rodent models of brain ID [79, 81].

##### Zinc deficiency

Zinc deficiency (ZD) is a micronutrient deficiency observed throughout the world (affecting ~ 31% of the global population) and is particularly prevalent in low-income countries [85]. Zinc is essential for the activity of hundreds of enzymes and for the functioning of the immune system [86]. As such, ZD has been shown to contribute to more frequent infections. It is also essential for supporting normal growth and development during pregnancy and childhood and has been associated with low birthweight and premature birth [87, 88]. It is estimated that 82% of pregnant women have inadequate zinc uptake worldwide [89, 90]. Despite its importance, there is a limited number of studies investigating the metabolic consequences of zinc restriction in early-life. Similarly, there is a lack of metabolic profiling studies investigating the other main micronutrient deficiencies highlighted by the WHO. This includes vitamin A, iodine and vitamin D and calcium deficiencies. One study explored the impact of early-life ZD on the gut microbiota and metabolome of mice using 16S rRNA gene sequencing of feces and ^1^H NMR spectroscopy of urine [59]. Zinc restriction had negligible effects on the gut microbiota but perturbations were seen in the urinary metabolites. The most notable were reductions in the excretion of BCAA catabolites consistent with the promotion of protein degradation in muscle. The excretion of 2-oxoglutarate, NMND, nicotinamide-*N*-oxide and hexanoylglycine was reduced in the ZD mice indicating a down-regulation of carbohydrate metabolism and lipid oxidation. Zinc is a co-factor for a range of enzymes involved in these processes.

##### Vitamin B_12_ deficiency

The impact of maternal vitamin B_12_ deficiency on the growth and hepatic metabolism of the offspring was explored by Roman-Garcia et al. [91]. Dietary vitamin B_12_ absorption requires gastric intrinsic factor (Gif). A Gif−/− mouse model was used to induce vitamin B_12_ deficiency and generate first- and second-generation offspring. Based on the hepatic metabolic profiles, B_12_ deficiency lowered the tissue abundance of metabolites involved in amino acid, choline, bile acid, protein, and purine metabolism. The most significant perturbations were found in taurine and hypotaurine metabolism highlighting taurine as a potential biomarker of B_12_-deficient status.

##### Vitamin D deficiency

Wang et al. applied a metabonomic approach in 200 children (6–38 months) to identify urinary biomarkers for the non-invasive diagnosis of nutritional rickets, a disorder commonly caused by vitamin D deficiency [92]. These researchers identified 31 biomarkers of nutritional rickets with the combination of phosphate and sebacic acid providing an accurate diagnostic measure. Several metabolic pathways, including ascorbate and aldarate metabolism, pentose and glucuronate interconversions, taurine and hypotaurine metabolism, calcium metabolism, and fatty acid oxidation, were modulated with nutritional rickets, providing novel insights into the pathogenesis and pathophysiology of the disease. Vitamin D has also been linked to a number of adverse pregnancy outcomes through unknown mechanisms. Finkelstein et al. studied the role of vitamin D status in serum metabolic profiles in 30 pregnant adolescents (13–18 years), 50% of which had low vitamin D levels [93]. Eleven metabolites differed based on vitamin D availability. These were involved in inflammation, oxidative stress, fatty acid oxidation, and gut microbial metabolism. Consistent with the findings of Wang et al., sebacic acid was also changed with vitamin D deficiency.

##### Vitamin E deficiency

Vitamin E refers to two families of compounds, the tocopherols and tocotrienols. These compounds are antioxidants and as they are only synthesized in plants they are essential nutrients. As the specific role of vitamin E in metabolic processes has not been defined, Moazzami et al. applied a metabolomic approach to investigate the effects of vitamin E deficiency on the hepatic metabolism of rats weaned onto either an α-tocopherol-deficient or an α-tocopherol-sufficient diet for 2 months [94]. Multivariate statistical analysis revealed that vitamin E deficiency caused an increase in carnitine, choline, valine, lysine, tyrosine and inosine levels and a reduction in glucose and UMP. Such changes indicate impaired protein synthesis, methyl donor status and a shift in energy metabolism. The same authors pursued this further by including γ-tocopherol and two additional groups: a marginal-deficient diet group and a fortified diet group [95]. Consistent with previous findings, vitamin E deficiency reduced glucose, but increased the abundance of choline-related metabolites (phosphocholine and betaine) and creatine in the liver, suggesting an alteration to cellular energy homeostasis. These alterations have been confirmed in zebrafish embryos over 5 days of development [96]. At 55 days post-fertilization, adult zebrafish were randomly allocated to an α-tocopherol deficient or an α-tocopherol sufficient diet for a minimum of 80 days up to 9 months. Vitamin E deficiency increased lipid peroxidation in the embryos, depleting phosphatidylcholine, choline and glucose resulting in lethal outcomes. The consistency in the alteration of metabolites involved in one-carbon metabolism, the primary mechanism underlying DNA methylation, could have epigenetic consequences and lead to long-term effects.

## Conclusions

Collectively, these studies illustrate the power of metabolic phenotyping to illuminate the diverse and dynamic biochemical perturbations induced by nutritional exposures and deficiencies throughout early-life. These methods allow the metabolic processes from the genome and also those from the gut microbiome to be studied simultaneously, providing an overview of the complete supra-organism and its response to nutritional modulations. Moreover, the biochemical fate of dietary components and their downstream impact on the biochemistry of the individual can be characterized helping to assess the impact of interventions and guide the development of novel strategies. Furthermore, as the metabolic phenotype of the individual represents a snapshot of their current metabolic state and their adaptations to nutritional restrictions there is great potential to tailor nutritional interventions to this information. This personalized approach to nutritional rehabilitation may help to improve the efficacy of treatments and reduce their adverse effects. Although metabolic phenotyping platforms are unlikely to be commonly available in developing countries, the development of smaller diagnostic or prognostic biomarker panels may facilitate simplified approaches for use in the field to guide interventional strategies. The increasing use of systems biology approaches, including metagenomics, genomics, methylomics, transcriptomics, proteomics, and metabonomics, is enabling the multiple disruptions induced by early-life malnutrition to be studied at several tiers of biomolecular organization allowing their collective impact on the complex mammalian system to be defined.
